# An Optically Pumped Magnetometer Working in the Light-Shift Dispersed *M*_z_ Mode

**DOI:** 10.3390/s17030561

**Published:** 2017-03-10

**Authors:** Volkmar Schultze, Bastian Schillig, Rob IJsselsteijn, Theo Scholtes, Stefan Woetzel, Ronny Stolz

**Affiliations:** 1 Leibniz Institute of Photonic Technology, Albert-Einstein-Straße 9, D-07745 Jena, Germany; bastian.schillig@yahoo.de (B.S.); theo.scholtes@leibniz-ipht.de (T.S.); stefan.woetzel@leibniz-ipht.de (S.W.); ronny.stolz@leibniz-ipht.de (R.S.); 2 Supracon AG, An der Lehmgrube 11, D-07751 Jena, Germany; rob.ijsselsteijn@supracon.com

**Keywords:** magnetometer, optically pumped magnetometer, light shift, *M*_z_, magnetic-field resolution, Latin Hypercube Sampling, Downhill simplex

## Abstract

We present an optically pumped magnetometer working in a new operational mode—the light-shift dispersed *M*_z_ (LSD-Mz) mode. It is realized combining various features; (1) high power off-resonant optical pumping; (2) *M*_z_ configuration, where pumping light and magnetic field of interest are oriented parallel to each other; (3) use of small alkali metal vapor cells of identical properties in integrated array structures, where two such cells are pumped by circularly polarized light of opposite helicity; and (4) subtraction of the *M*_z_ signals of these two cells. The LSD-Mz magnetometer’s performance depends on the inherent and very complex interplay of input parameters. In order to find the configuration of optimal magnetometer resolution, a sensitivity analysis of the input parameters by means of Latin Hypercube Sampling was carried out. The resulting datasets of the multi-dimensional parameter space exploration were assessed by a subsequent physically reasonable interpretation. Finally, the best shot-noise limited magnetic field resolution was determined within that parameter space. As the result, using two 50 mm^3^ integrated vapor cells a magnetic field resolution below 10 fT/√Hz at Earth’s magnetic field strength is possible.

## 1. Introduction

Magnetic field measurement is an important issue in various fields of science. This covers Earth’s field mapping in ground-based [1] or spaceborne [2] geomagnetic measurements and in archaeological prospection [3,4] as well as biomagnetic field measurements in medicine [5,6] or investigations in fundamental physics [7,8]. Besides this, many tasks cover routine applications, for example in automotive systems [9]. Correspondingly, diverse types of magnetometers exist [10]. However, for all measurement cases, where highest possible magnetic field resolution is needed, for a long time Superconducting Quantum Interference Devices (SQUIDs) were the only choice. SQUIDs can reach noise-limited magnetic field resolutions of *B*_n_ < 1 fT/√Hz [11,12], but need cryogenic cooling for operation Therefore, also optically pumped magnetometers (OPMs) [13] have been used in magnetometry since several decades [14]. OPMs are based on the interaction of atomic spins with an external magnetic field *B*_0_ to be measured [15,16]. In the configurations regarded here, the atoms of an alkali vapor are polarized by optical pumping, this means their spins are oriented in the propagation direction of the pumping light, which is tuned to an absorption line of the gas and which is circularly polarized [17]. Already in one of the very earliest publications on optical magnetometers, two basic configurations were presented [18]: the *M*_z_ and the *M*_x_ mode. In the *M*_z_ mode, a change in DC light absorption is observed. It has best resolution when magnetic field and pumping beam are parallel to each other. In the *M*_x_ mode they are inclined by about 45°. Also the *B*_1_ field in resonance with the Larmor frequency *f*_L_ of the atomic spins [19,20] needs different orientation (parallel to the pumping beam for *M*_x_ and perpendicular for *M*_z_). In the *M*_x_ mode, the transmitted light is modulated at the *B*_1_ field frequency. For that reason this configuration became prominent during further development, because it offers the possibility to use a lock-in configuration, which allows signal detection at the *B*_1_ field frequency and thus avoids low-frequency noise contributions from technical sources (pump laser, pre-amplifier, feedback electronics).

A central aim of any magnetometer development is the improvement of magnetic field resolution. The intrinsic noise-limited magnetic field resolution *B*_in_ of optically pumped magnetometers is determined by the spin-projection noise [16,21]:

(1) $$B_{\mathrm{in}}=\frac{1}{2\pi \cdot \gamma }\sqrt{\frac{\Gamma }{nV}}$$

Here γ is the gyromagnetic ratio of the alkali atom used (3.5 kHz/μT for Cs). *B*_in_ depends on the vapor density *n* of the alkali atoms and on the volume *V* of the vapor cell. For that reason, for a long time the use of large vapor cells was the silver bullet for high resolution [19,22]. Recently, also the opposite has been tried, because smaller alkali vapor cell volumes offer the possibility of integration with other functional parts of the complete magnetometer (like pump laser diodes and photo detectors [23]) or the combination of various cells with identical properties [24], which can be used for signal improvement [25,26]. Moreover, small cells are a good choice for measurements, where an adequate lateral resolution is requested, like in biomagnetism [27]. In order to achieve competitive magnetometer resolution despite a small cell volume, small relaxation rates Γ are required. With properly designed vapor cells, relaxation due to collisions of the alkali atoms with the walls and with buffer gas molecules can be made small enough, so that spin-exchange collisions between the alkali atoms themselves become the dominant relaxation mechanism. A prominent solution for the suppression of this spin-exchange relaxation, found by Kominis et al. [28], is the SERF magnetometer (SERF = spin-exchange relaxation-free), where very high atomic density *n* is combined with very low ambient magnetic field magnitude *B*_0_. These SERF magnetometers offer an ultimate resolution in the 1 fT/√Hz range for active cell volumes of about 100 mm^3^. They have become interesting for investigations of human heart or brain activities with magnetocardiography (MCG) and magnetoencephalography (MEG) [27,29]. The drawback of the SERF OPM is their restriction to near-zero environmental magnetic field strengths, calling for expensive magnetic shielding and making them inadequate for measurements within the Earth’s magnetic field.

The intrinsic noise-limited magnetic-field resolution *B*_in_ represents the ultimate resolution value. In the majority of cases, however, this is overwhelmed by the photon shot-noise limitation *B*_sn_ of the magnetic-field resolution, given by:

(2) $$B_{\mathrm{sn}}=\frac{I_{\mathrm{sn}}}{\gamma \cdot \left|dI_{d}/df_{B1}\right|}$$

It depends on the photon shot-noise of the detected laser light:

(3) $$I_{\mathrm{sn}}=\sqrt{2eI_{\mathrm{dc}}}$$

which in its turn is determined by the dc photo current *I*_dc_ generated in the photo diode behind the vapor cell. $\left|dI_{d}/df_{B1}\right|$ is the steepness of the dispersive signal around the Larmor frequency.

In this paper we present a newly developed operational mode of OPM which we named Light-Shift Dispersed *M*_z_ (LSD-Mz) mode. Such LSD-Mz OPM is able to work in Earth’s magnetic field strength (about 50 μT in Central Europe) and at the same time has the capability to reach down to the 10 fT/√Hz magnetic field resolution range. The performance of the magnetometer depends on a number of operational parameters. The complete analytical description of their interactions would require advanced and very cumbersome quantum physics modelling. Also an exhaustive experimental search for the parameter set for best shot-noise limited magnetic-field resolution *B*_sn_ would be inadmissibly laborious. For that reason we decided to build up an approximation model over the complete available parameter range based on a manageable sample data set of evaluable measurements, acquired by means of Latin Hypercube Sampling.

The content of the next chapters is as follows: in a first part we describe the new LSD-Mz magnetometer—its genesis from preceding OPM types, the experimental setup for its characterization, and the dependence of the LSD-Mz properties on the most important operational parameter, the pump laser power. In the second part the complete characterization is presented, after the optimization strategy and its experimental realization are explained. The paper is concluded with an evaluation of the results and an outlook.

## 2. The LSD-Mz Magnetometer

### 2.1. Genesis of the LSD-Mz OPM

**Figure 1:**
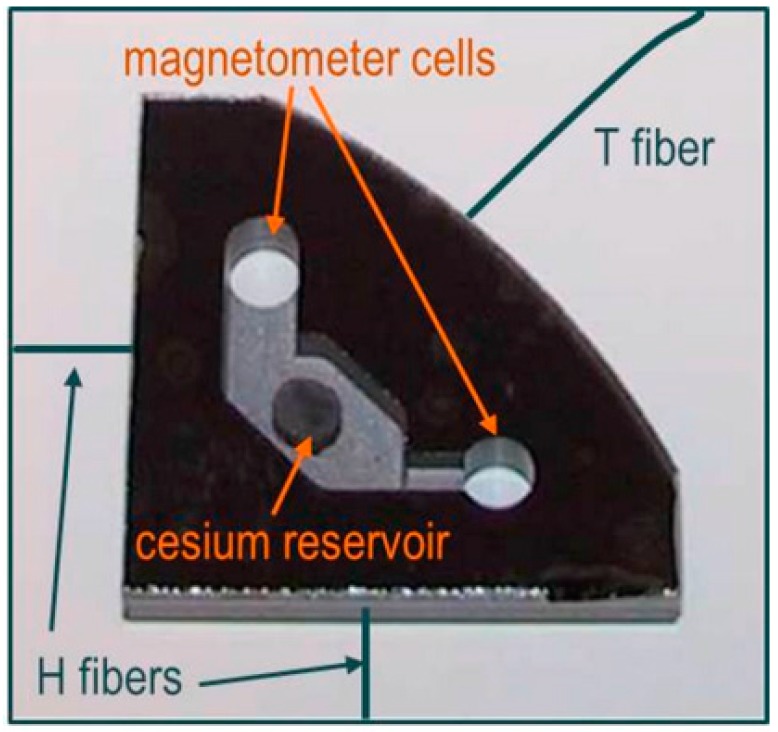
Photograph of an integrated configuration of two magnetometer cells, both connected to a common cesium reservoir. Fibers for heating this cell array (H fibers) and temperature measurement (T fiber) are sketched (reproduced with permission from Schultze [31], copyright 2015 Optical Society of America).

All investigated OPM types presented here used the same 4 mm diameter magnetometer cells, integrated in a 4 mm thick silicon wafer, what corresponds to a cell volume of 50 mm^3^. The cell assembly is shown in Figure 1. The integration with a common cesium reservoir ensures identical operational conditions of the cells. The fabrication of this cell arrangement follows a procedure described elsewhere [30]. During the cell production, the cells are filled not only with cesium as the active medium, but also with an appropriate amount of nitrogen buffer gas. 

**Figure 2:**
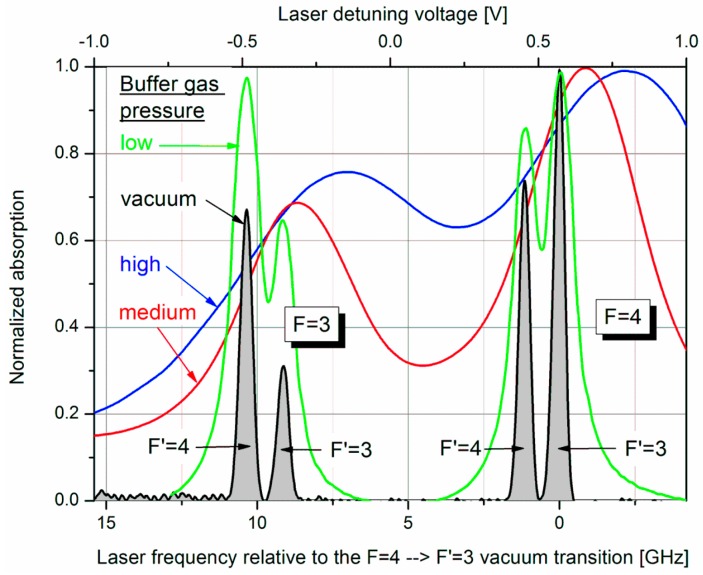
Absorption spectra of Cs cells with different N_2_ buffer gas pressure. For the determination of the respective pressure the absorption spectra were fitted by Lorentz profiles. Using the data from Couture [32], broadening as well as shifting of the absorption lines with reference to a vacuum cell delivered consistent values of 20, 125, and 250 mbar at 130 °C for low, medium, and high pressure, respectively. All absorption curves are normalized to their individual maximum.

Besides the common benefits of this buffer gas (quenching and hindering of cesium atoms’ movement to the walls in order to suppress relaxation on the walls) we make use of its broadening effect on the absorption profiles of the optical transitions, which, for example, enables the simultaneous pumping to both excited states (cf. Figure 2) using a single narrow-band laser.

The starting configuration of our developments was the *M*_x_ mode. Only one channel was used. This integrated magnetometer delivered values of *B*_sn_ around 200 fT/√Hz. Depending on the buffer gas pressure we measured 265 fT/√Hz [33] and 173 fT/√Hz [31] for low (20 mbar) and high (250 mbar) nitrogen buffer gas pressure, respectively.

The effect of higher buffer gas pressure was one of the features in the subsequent operational mode, called “Light-Narrowing (LN)” [34]. As can be seen in Figure 2, the higher the buffer gas pressure, the more the four absorption lines are broadened and overlapped. Most importantly, for the highest pressure this even holds for the ground-state levels to a remarkable amount. For this high pressure several more changes were made by the transition from normal *M*_x_ mode to the LN one:

1. The pump laser frequency was tuned from the commonly used F = 4 transitions to the F = 3 transitions.
2. The laser power *P*_L_ was increased by more than one order of magnitude (from about 0.5 mW to about 20 mW).
3. The cell temperature was slightly increased (from about 100 °C to about 120 °C).

**Figure 3:**
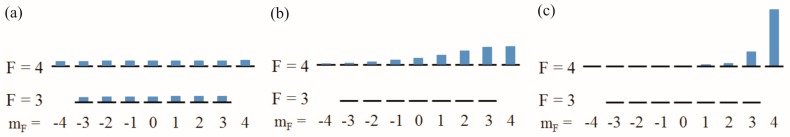
Populations of the Cs ground states for cells with high buffer gas pressure, calculated with the formalism developed by Scholtes et al. [35]: (**a**) in conventional *M*_x_ mode operation (pumping on F = 4); (**b**) in LN *M*_x_ configuration (pumping on F = 3 with about 50-fold overall pumping rate); and (**c**) in LSD-Mz configuration. (In contrast to the earlier published ones [34] these ground-state populations take into account the high buffer gas pressure and especially the redistribution of populations from the outer Zeeman levels back to the inner ones due to the 45° inclined *B*_0_-field with respect to the pump beam direction in Figure 3a,b.

This way, the pumping rate on the F = 4 ground state remains similar compared to the normal *M*_x_ mode, but the F = 3 ground state is strongly depleted due to the strong pumping. This can be seen in Figure 3a,b which show the population of the Zeeman levels in both ground-states levels for the normal and the LN *M*_x_ mode. In consequence, all atoms contribute to the measurement signal, what increases the steepness $\left|dI_{d}/df_{B1}\right|$ of the dispersive lock-in signal by two orders of magnitude [34]. The same effect can be achieved by substituting the single detuned laser by separate pumping on F = 4 and repumping on F = 3 [31,35], a common scheme in cold atom experiments [36].

The LN mode finally reflects in a very much improved shot-noise limited magnetic-field resolution *B*_sn_ of about 40 fT/√Hz. Yet, the LN mode also has another, critical side-effect. Strong pumping with circularly polarized light originates a remarkable magnetization of the alkali vapor in the direction of the pumping light. Moreover, detuned pumping (detuned from F = 4) generates a strong light shift [37], viz. a virtual magnetic field in the direction of the pumping light. This field adds vectorially to the measurement field *B*_0_ and the magnetic-field measurement is falsified. Since the direction of the light shift is opposite for different helicity of the circularly pumped light, the Larmor frequency is shifted either to lower or higher values.

**Figure 4:**
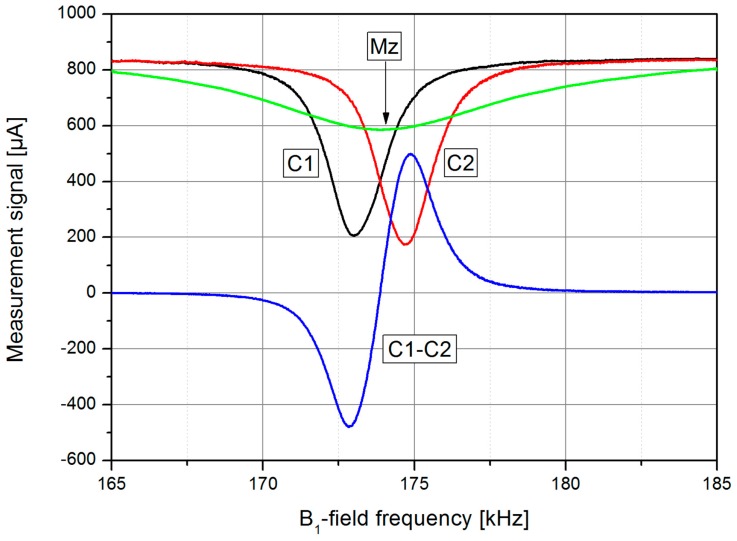
*M*_z_ signals of two single cells (C1 and C2) pumped in the LN mode with different helicity of the circularly polarized light and LSD-Mz difference signal (C1-C2), in dependence on the *B*_1_-field frequency (laser pumping power *P*_L_ = 1.5 mW, cf. later). A conventional *M*_z_ signal (Mz) with pumping on F = 4 is shown for comparison. The *B*_0_-field had a value of about 50 μT.

The new LSD-Mz mode overcomes these constraints, it even benefits from these effects. This OPM works in the *M*_z_ mode, where *B*_0_-field and pump laser direction are parallel. In this case, using the off-resonant pumping technique described above, nearly all atoms can be pumped into the stretched state, the Zeeman state with the highest quantum number (Figure 3c). This enhances the signal further and also decreases spin exchange relaxation due to the light-narrowing phenomenon [34,38]. However, according to Bloom [18] the measured variable in the *M*_z_ mode is just a secular change of the pumping light absorption, when the *B*_1_-field works at the Larmor frequency. As can be seen in Figure 4, this effect is weak for normal working conditions, i.e., pumping at F = 4. Using the LN conditions, this signal depth can be increased remarkably up to about 75% of the photo signal. Additionally, due to the light shift the resonance frequency is strongly shifted. Now we use the signals of two cells (C1 and C2) which are pumped with opposite helicity, resulting in an opposite shift of the resonance frequencies. The simple difference of these two dc signals has a neat dispersive character, thus can easily be used for the measurement of the mean frequency. Due to the parallel and anti-parallel orientation of the light shift with respect to *B*_0_ this frequency corresponds to the Larmor frequency. With growing angle between *B*_0_ and light shift their vectorial addition results in a slightly increasing falsification of the Larmor frequency, which in the LN *M*_x_ configuration with its best sensitivity in 45° direction cannot be eliminated completely anymore. Beside this advantage of the LSD-Mz mode, the pure dc measurement has the additional positive side-effect, that the crucial problem of tuning the reference phase to the correct value in lock-in measurements is omitted.

### 2.2. Experimental Setup

**Figure 5:**
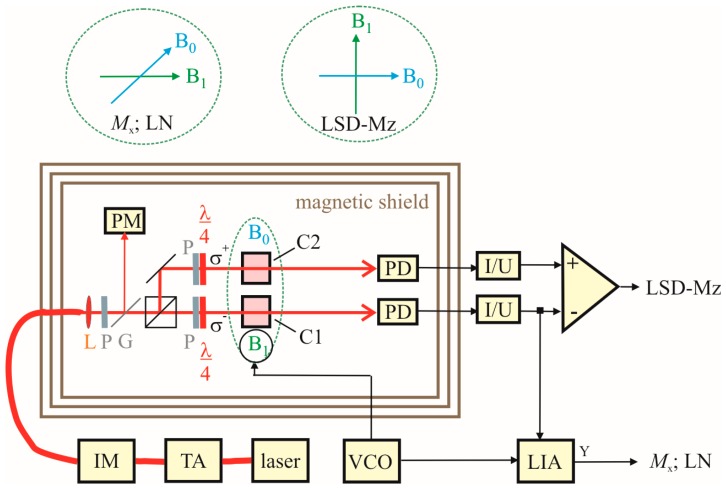
Measurement setup. Two integrated cesium cells C1 and C2 (in the center of the magnetic shielding), the optical elements (lenses (L) for beam collimating, linear polarizers (P) and quarter-wave plates (λ/4) for the creation of circularly polarized light), and photodiodes (PD) for signal detection are located inside a shielding barrel with Helmholtz coils (not shown) for *B*_0_ and *B*_1_ field generation. The pump laser beam (amplified by a tapered amplifier (TA) and tuned by an intensity modulator (IM)) as well as the heating laser beam (not shown) are both fed into the shielding barrel via optical fibers. The pump power reference value (calibrated to those at the cells) is split off by a glass plate (G) and measured with a power meter (PM). The two PD currents (amplified by trans-impedance amplifiers (I/U)) and their difference deliver the actual measurement signals. The *B*_1_ field is provided by a voltage-controlled oscillator (VCO). The different directions of *B*_0_ and *B*_1_ field in the *M*_x_, Light-Narrowing (LN), and LSD-Mz mode are sketched separately. For *M*_x_ and LN only channel 1 is used with lock-in amplifier (LIA) signal processing, for LSD-Mz the difference of the signals from channel 1 and 2 is used.

The measurement setup for the OPM characterization is sketched in Figure 5. The cells are pumped by a DBR laser diode with wavelength of λ = 894.6 nm, followed by a tapered amplifier. The latter was implemented in order to have enough power available, so that the pump laser power could be tuned by an intensity modulator (realized by a Mach-Zehnder interferometer using integrated lithium-niobate waveguide structures) without altering the operational parameters of the laser diode and the tapered amplifier. This ensures stable operation and avoids unwanted detrimental simultaneous changes of other laser parameters. The pump laser radiation is split into two beams of equal power for the two cesium cells. In the separated beams, packages of polarizers and quarter-wave plates fine-tune the pump powers in the two cells and provide σ^+^ and σ^−^ circularly polarized light. The cell array is temperature-controlled by off-resonant (λ = 976 nm) heating laser radiation, fiber-coupled to the face sides of the silicon wafer. This setup is located inside a three-layer μ-metal shielding barrel of 1 m in diameter and 1 m length. The measurement field with Earth’s field strength of *B*_0_ ≈ 50 μT is provided by large pairs of Helmholtz coils for the three orthogonal spatial directions. This guarantees a *B*_0_ field with homogeneity distortions less than 2 × 10^−3^ across the cell array [25]. The *B*_1_ field is generated by smaller rectangular Helmholtz coils of 10 cm side length. The various OPM operational modes described in the previous section need different orientations of pumping light, *B*_0_-field, and *B*_1_-field with respect to each other. This is also sketched in Figure 5. All measurement parameter variations are performed computer-controlled and the characteristics of the measured resonance curves are automatically extracted via Levenberg-Marquardt fits of the Lorentz curves.

### 2.3. LSD-Mz Parameters vs. Pump Laser Power

For the first investigation of the LSD-Mz magnetometer a set of operational parameters which gave the best results in the LN mode was used: pumping centrally at the F = 3 absorption line, 125 mbar nitrogen buffer-gas pressure, 128 °C operation temperature, and 236 nT_rms_ *B*_1_-field strength.

The laser pumping power *P*_L_ has very prominent influence on all the parameters determining the shot-noise limited magnetic-field resolution, because *P*_L_ has direct impact on the photon shot-noise, on the magnetic resonance curve parameters, and (via the light shift) on the relative shift of the two magnetic resonance curves to each other. This interplay and its impact on the magnetometer parameters are investigated more in detail and shown in the subsequent figures. 

**Figure 6:**
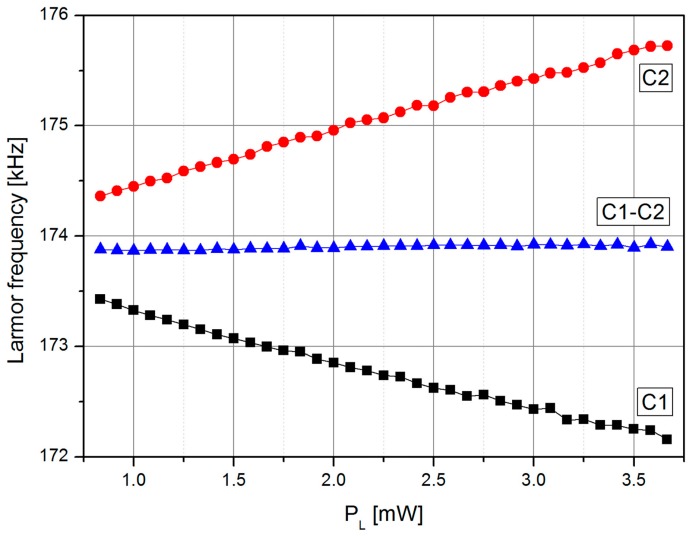
Dependence of the resonance frequencies of the two single cells and the zero crossing frequency of the LSD-Mz signal on the laser pumping power *P*_L_.

Figure 6 shows the dependence of the individual cells’ resonance frequencies and the position of the zero crossing of the LSD-Mz signal on the laser power. The results confirm the intended suppression of light-shift induced falsification of the two individual cell signals as can be seen in the resulting zero crossing of the difference signal. In case of a magnetic field gradient across the cell assembly, the difference signal would represent the mean value of the magnetic fields at C1 and C2.

**Figure 7:**
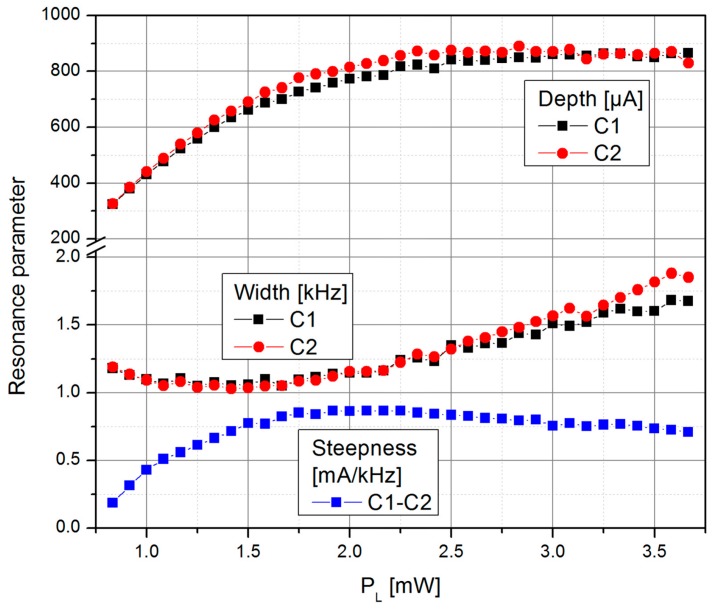
Dependence of the resonance width (half width at half maximum), determined by the relaxation rate Γ, and depth of the two individual cells and the resulting LSD-Mz signal steepness d*I*_d_/d*f*_B1_ on the laser pumping power *P*_L_.

The signal steepness (Figure 7) is the result of the changing individual resonance parameters (width and height) of the two cells and the splitting of the individual resonances caused by the light shift effect (Figure 6). 

**Figure 8:**
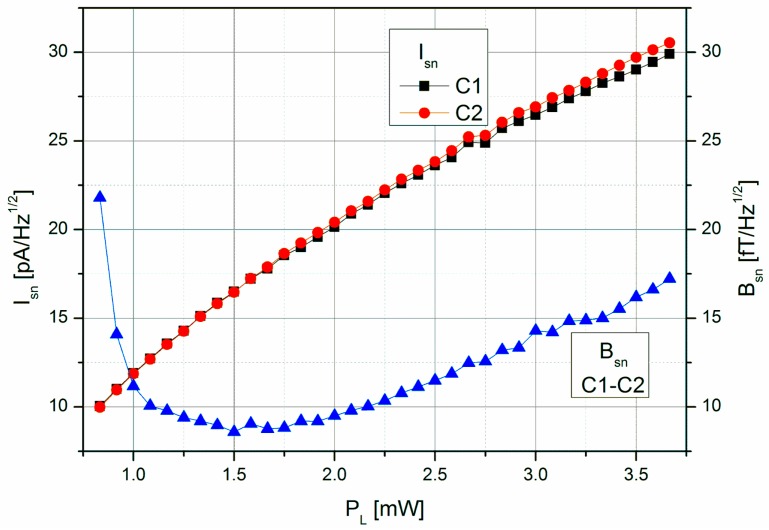
Dependence of the photo-current shot-noise *I*_sn_ of the individual cells and the shot-noise limited magnetic field resolution *B*_sn_ of the LSD-Mz magnetometer on the laser pumping power *P*_L_.

Figure 8 shows the shot-noise limited magnetic field resolution of the LSD-Mz magnetometer, resulting—according to Equation (2)—from the signal steepness and the photo-current shot-noise of the two cells (taking into account the increase by a factor of √2 as the detected photo current doubles with the use of two cells). The optimum value of *B*_sn_ ≈ 9 fT/√Hz represents a remarkable improvement compared to the 40 fT/√Hz in pure LN operation of the same cell [26,31].

The superior magnetic field resolution of the LSD-Mz magnetometer mainly results from the large resonance signal depth, while the resonance signal width still remains in the 1 kHz range (we measured 3 dB bandwidths of 600 Hz and 1 kHz without and with feedback regulation of the *B*_1_-field frequency, respectively). Such large resonance width is advantageous since for *M*_z_ magnetometers it directly translates to the sensor bandwidth [39]. 

## 3. Complete LSD-Mz Parameter Characterization

### 3.1. Optimization Strategy and Experimental Realization

The investigation of the complete LSD-Mz parameter set was performed with the help of optiSLang [40], a software platform developed by Dynardo GmbH (Weimar, Germany). As opposed to its original field of application—virtual product development—optiSlang was used here for the first time to control and optimize a real complex system in operation.

**Figure 9:**
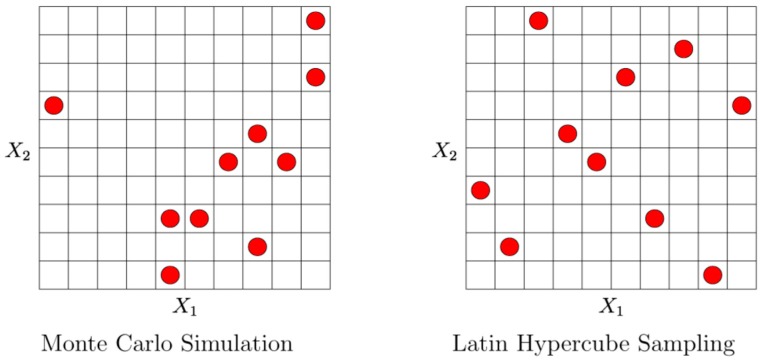
Monte-Carlo method vs. Latin Hypercube Sampling in 2D.

OptiSLang is based on Structural Language (SLang) which provides a tool set for optimization and reliability analysis in finite element simulations. With global variance-based sensitivity analysis, not the input variable influence at a local sample point is evaluated, but rather the variation of the system response is apportioned to the variation of input variables over the full range of parameters [41]. In our case we looked at how pump laser power and pump laser wavelength as well as cell temperature and *B*_1_-field amplitude influence the output values of the magnetometer system. Here sensitivity analysis served the purpose of issuing a global system behavior model in relation to the parameters given above. It identifies important design variables, mechanisms and relations in the system. It is also useful in assessing the importance of each input value with respect to the final result. If found unimportant, an input variable can be stripped from the behavioral model for simplification and improvement of the prediction quality of the global model. The full-factorial coverage of the design space by serial single sweeps of input parameters requires a large amount of samples, including the downturn of redundant measurements when a previously acquired parameter combination occurs again. For that reason, a stochastic sampling approach was chosen in form of the Latin Hypercube Sampling (LHS). This method is based on the separation of the value range of any given distribution into *N* intervals, and the extraction of a random sample per interval (*N* = number of random samples). This ensures the complete coverage of the value domain for each variable. Figure 9 compares Latin Hypercube Sampling with the Monte-Carlo random sampling method in two dimensions, showing the clear advantage of LHS. Notice the uniformity of distribution in the right picture, with one sampling point for every row and column, where with purely random Monte-Carlo, columns are left out where on the other hand, there are clusters of samples close together. Such clustering as observed in Figure 9 (left) may lead to falsely suggested input correlations to an approximation model where none are present. To account for this, optiSLang uses a strategy of advanced LHS, where these unwanted correlations are strongly minimized by optimization [42,43].

For sampling over the full available magnetometer parameter range, a LHS sample size of 60 discrete sampling intervals was chosen. This way, every 1 °C step from 80 °C to 140 °C temperature range was measured while avoiding interchanged sample assignment potentially resulting from temperature sensor inaccuracy (±0.3 °C). To cover the probability of failed designs (where the parameter combination is outside the LSD-Mz working range), ten LHS sample designs were combined for measuring each Cs vapor cell. This way, each temperature step was measured in ten equally distributed random combinations of the other three parameters. Requiring a total of 600 measurements per cell, with one measurement taking approximately one minute, the sensitivity analysis of one cesium cell would take about ten hours of measurement time. This is very reasonable compared to the effort of a full factorial sampling of 60 steps, where 60^4^ min (24 years) would be needed to acquire each possible combination of input parameters.

The forecast quality of an approximation model deduced from the above measurements is expressed in optiSLang by the Coefficient of Prognosis (CoP), rather than only interpreting how good the approximation model fits through the sample points. It is a model-independent measure and is calculated by:

(4) $$CoP=1-\frac{SS_{E}^{pred}}{SS_{T}}$$

where $SS_{E}^{pred}=\sum_{i=1}^{N}{\left(y_{i}-{\hat{y}}_{i}\right)}^{2}$ is the error sum of squares of a prediction model, and $SS_{T}=\sum_{i=1}^{N}{\left(y_{i}-\mu_{\Upsilon }\right)}^{2}$ is the total variation of the measured output data Υ. *y*_i_ are the sample values, and ${\hat{y}}_{i}$ and $\mu_{\Upsilon }$ are the value predicted by the approximation model and the mean value of the measured data, respectively. Using CoP, a Metamodel of Optimal Prognosis (MOP) replaces the scanned model responses from sensitivity analysis by mathematical descriptions for each response. During the MOP generation process, different meta-model types were tested on the sample data. To evaluate the model’s quality and predictive ability, the model-explainable variance was estimated by cross validation. By using various techniques to map the complexity of the response behavior (like linear regression, moving least squares, response surface method, isotropic kriging, support vector regression or artificial neural networks), the best suited technique was chosen based on its performance regarding to the CoP. Once the optimal modelling technique was determined, the importance of input variables was estimated using Sobol indices. Variables of very low or no importance were stripped from the model base, further improving approximation quality.

Based on the performance of the real technical set-up, two kinds of final optimization were carried out to give the optimal parameter combination for the best shot-noise limited magnetic-field resolution *B*_sn_. Both optimization methods started from the best parameter combination for low *B*_sn_ measured in sensitivity analysis. The first method is called the Adaptive Response Surface Method (ARSM), a single-objective optimization approach based on linear or quadratic polynomial approximation of the measured data. Starting from the initial value, in its surrounding with a start range α, experiments were carried out. From a polynomial fit of the results, the minimum was determined which served as starting point for the next approximation with shrunken range α [44]. The second optimization method, Downhill simplex, is based on an algorithm proposed by Nelder and Mead [45]. It is well suited for solving nonlinear optimization problems with a single target function in multidimensional space. It is based on morphing a simplex geometrical shape, a polytope of *n* + 1 points in *n* dimensions. 

Since the optimization of the LSD-Mz magnetometer was performed on real samples, the optiSLang software selected the operational parameter constellations to be measured. Via an inter-program ASCII interface optiSLang communicated with LabView measurement routines which set the measurement conditions. Four of the five operational parameters were statistically chosen due to the Latin Hypercube Sampling (LHS); the *B*_1_-field strength, the pump laser power *P*_L_ and its wavelength (which in atomic physics is usually expressed by differences in laser frequency), and the cell temperature *T*_cell_. The fifth parameter, the nitrogen buffer gas pressure *p*_N2_, though being an input parameter for optimization in a physical sense, contrary to the other input parameters was not variable within one optimization run, as it is determined by the cell used. Instead, two cells with different buffer gas pressures were optimized separately.

### 3.2. Investigation of the Complete LSD-Mz Parameter Set

In order to cover the complete operational parameter set, the magnetometers were investigated within the following parameter ranges:

- Two samples with buffer gas pressures *p*_N2_ = 125 and 250 mbar at 130 °C, respectively, were measured separately. They have been denominated as “medium” and “high” buffer gas pressure cells in Figure 2. In the following we will use these terms for the two magnetometers. The pump laser power dependence of the medium-pressure magnetometer was already shown in the preceding section.
- The *B*_1_-field strength was varied using an oscillator voltage between 0.1 and 4 V_rms_. With the *B*_1_-field coil constant of 347 nT/mA and 1 kΩ resistance this corresponds to a variation between 34.7 nT_rms_ and 1.39 μT_rms_. The lower boundary is where a resonance line barely can be detected. At the upper boundary the large resonance widths distorts the dispersive signal.
- The pump laser power was varied between 0.5 and 4.5 mW, based on the results obtained before (cp. Section 2.3).
- The cell temperature was investigated between 80 °C, the lower boundary, where a resonance signal could be detected, and 140 °C, the upper boundary, above which most of the pumping light is absorbed.
- The pump laser frequency is responsible for the relation which hyperfine transitions are more or less pumped (cf. Figure 2). For this variation, a dc voltage between −1 and +1 V is applied to the modulation input of the laser current driver, which translates to a laser frequency variation range of about 25 GHz, covering the absorption lines of the investigated magnetometer cells as shown in Figure 2.

**Figure 10:**
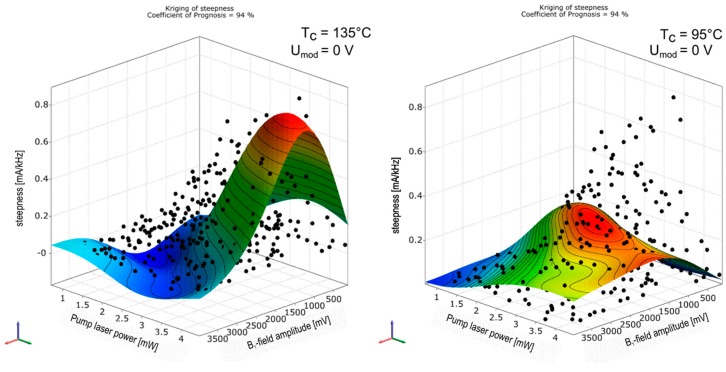
Response surface plot of steepness over the two input variables *B*_1_–field amplitude and pump laser power *P*_L_ for two different cell temperatures *T*_c_.

**Figure 11:**
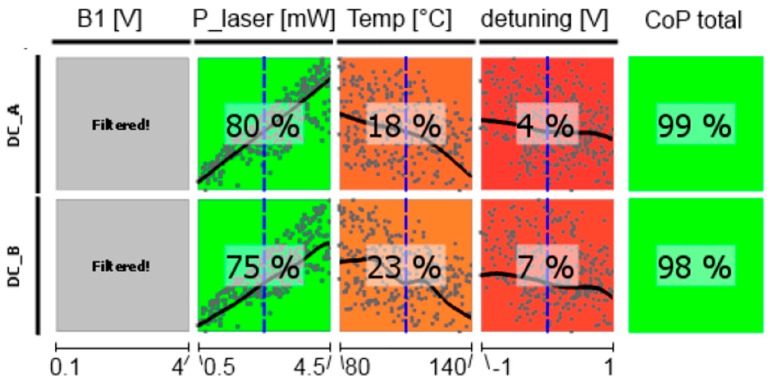
CoP matrix of the dc photocurrents of the medium-pressure magnetometer. Approximation technique: moving least squares (DC_A) and isotropic kriging (DC_B).

**Figure 12:**
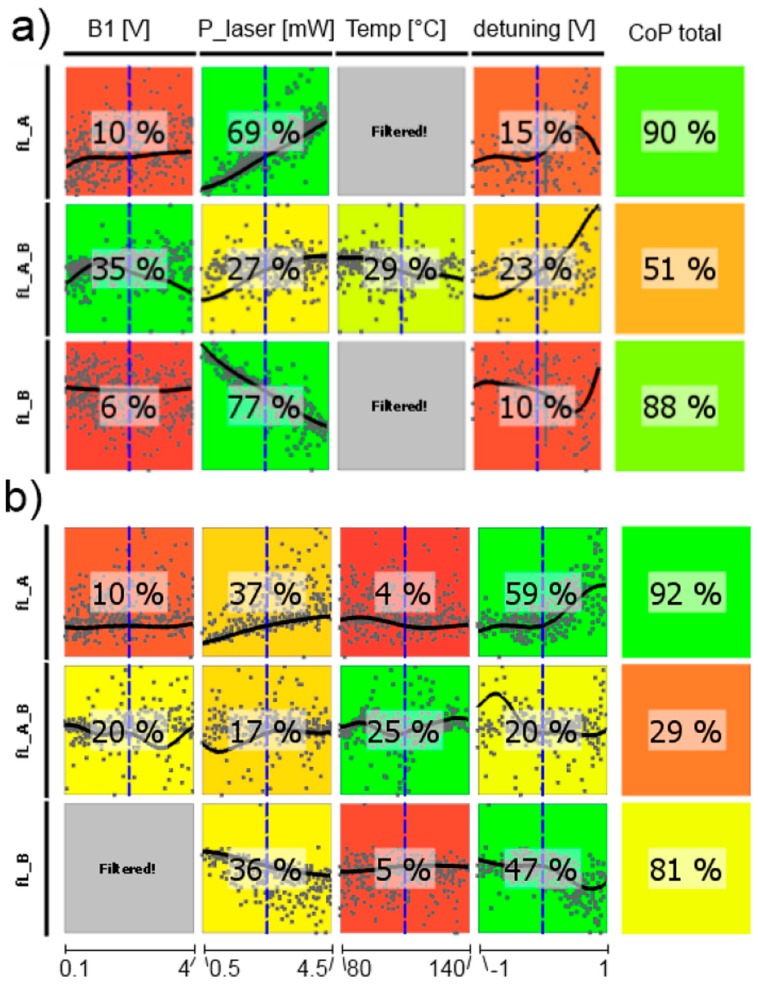
CoP matrix of the resonance frequencies. (**a**) high-pressure magnetometer; (**b**) medium-pressure magnetometer. Approximation technique: (**a**) fL_A moving least squares; all others isotropic kriging.

**Figure 13:**
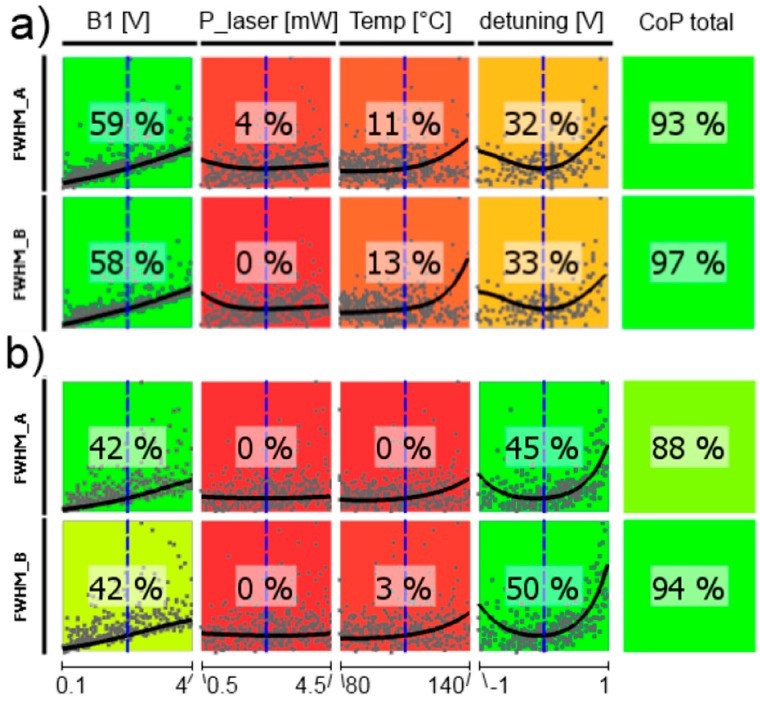
CoP matrix of the resonance widths. (**a**) high-pressure magnetometer; (**b**) medium-pressure magnetometer. Approximation technique: (**a**) isotropic kriging; (**b**) linear regression.

**Figure 14:**
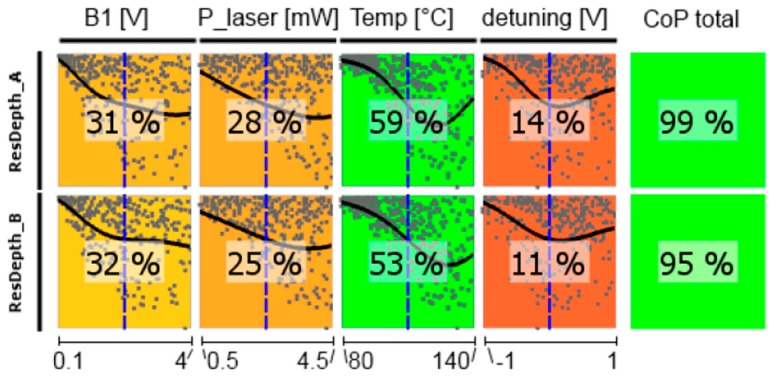
CoP matrix of the resonance depths for the medium-pressure magnetometer. Since the resonance depth is calculated as the difference between peak current and minus dc level, the values are negative. So, lower values means higher absorption. Approximation technique: isotropic kriging.

**Figure 15:**
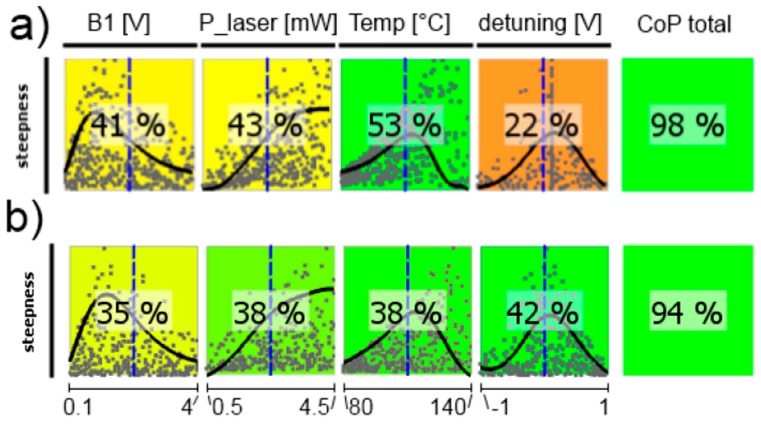
CoP matrix of the difference signals’ steepness near the Larmor frequency. (**a**) high-pressure magnetometer; (**b**) medium-pressure magnetometer. Approximation technique: isotropic kriging.

**Figure 16:**
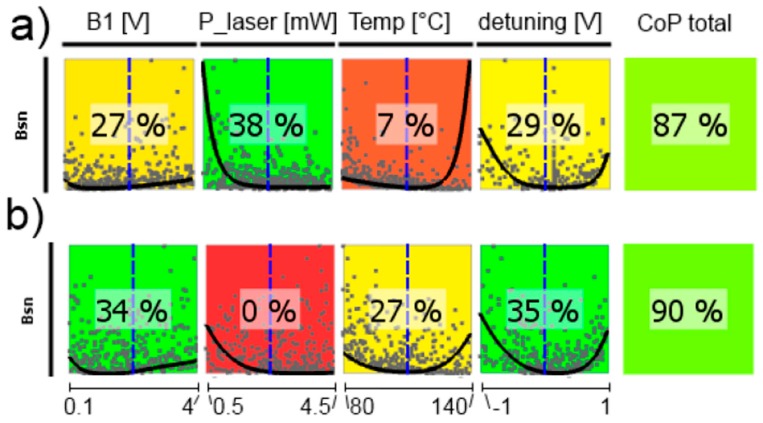
CoP matrix of the shot-noise limited magnetic-field resolution *B*_sn_. (**a**) high-pressure magnetometer; (**b**) medium-pressure magnetometer. Approximation technique: isotropic kriging.

The latter four technical input parameters are later used in the abscissas of Figure 11, Figure 12, Figure 13, Figure 14, Figure 15 and Figure 16. The determination of the magnetometer’s parameters with the formalism described above is exemplarily shown in Figure 10. The colored surfaces are the approximation model responses for the signal steepness in dependence on two input parameters—*B*_1_-field amplitude and pump laser power—with the other input parameters fixed at constant values. The black dots correspond to all measured values from the sensitivity analysis, where all four input parameters are varied. For that reason not all sample points are located on this response surface plot reduced to two inputs and one output parameter. Changing the constant values of omitted variables alters the course of the response surface plot in a way that it leads through their corresponding sample point as well, if the value fits the measured parameter combination. The two pictures in Figure 10 show plots in the same domain, with different cell temperatures. The response surface changes, while the black sample points remain unaltered.

In the following, CoP matrices of input parameter relevance for the respective outputs obtained by sensitivity analysis are discussed, where CoP is a quantification of the forecast quality of a meta-model for the prognosis of result value variation. These matrices (shown in Figure 11, Figure 12, Figure 13, Figure 14, Figure 15 and Figure 16) contain the following information: Each row depicts an output parameter in dependence on all input parameters, and each column shows the influence of a single input parameter on the chosen output. Each colored square presents a curve of a single input vs. a single output parameter, with all measured input combinations (where all input values are varied for each sample) as grey dots, and the approximation model response as the black line (where only the input in question is varied in the forecast model, with all other parameters fixed at the blue dashed vertical lines in the other squares). The number in the center specifies the CoP of the output achieved by interpreting the reduced data set of just the actual input alone. CoP—being based on variance—is a measure of how much an output value performance relies on the input being at the right value. The outermost right column specifies the total CoP achieved by the full approximation model. Using various approximation techniques mapping the complexity of response behavior, the best suited technique is chosen based on its performance regarding the total CoP. This technique is appointed in the respective figure caption. If the CoP values for individual inputs add up to more than 100%, this indicates that coupling terms exist in the model. If an input shows to be of very low or no significance to the output, the regarding variable is filtered from the meta-model, which is indicated by a grey square. Filtering is used if a higher total CoP can be achieved by the reduced model.

The dependence of the detected photocurrent behind the two cells of the LSD-Mz magnetometer (measured off the magnetic resonance) clearly shows the power of the CoP matrix (Figure 11). Pump laser power is of most significance, showing a nearly linear relation. Also temperature has an effect of lowering the current with higher values, because more cesium atoms are set free and available for absorption. Laser detuning imprints the absorption spectrum on the photocurrent (cp. Figure 2). Total CoP is almost 100%, thus the action of the dc currents is very well described by the meta-model. The figure shows the medium-pressure magnetometer. The results of the high-pressure one are identical. Due to Equation (3) also the photo current shot noise has completely the same dependencies and CoPs.

From the dependence of the resonance frequencies shown in Figure 12 first the single channels will be discussed. The total CoP is high, so the statements are reliable. Naturally, *B*_1_-field strength as well as cell temperature have negligible influence. The dependence on the laser pump power is inverse for cells A and B, which is due to the opposite light shift in these cells introduced by counter-oriented circular laser polarization. This inverse course also applies for the detuning, which reproduces the strength of the individual absorption lines—comparable to the optical absorption spectrum (cp. Figure 2). The influence of the detuning is stronger for the medium-pressure cell (CoPs are higher), because here the single absorption lines are more distinct than for the high-pressure cell. The resonance frequency of the difference signals should be independent of all operational parameters, thus always deliver the Larmor frequency. This was the basic idea of the LSD-Mz magnetometer (cp. also Figure 4 and Figure 6). Vanishing CoP values should reflect this. In fact these numbers are low, but not zero. This reflects the susceptibility of the magnetometer concept to a mismatch between the operational parameters of the two cells, what constitutes a severe demand on the proper experimental set-up of the complete magnetometer. 

The resonance widths are well described by the metamodel (high total CoPs in Figure 13). For both magnetometer cells only *B*_1_-field strength and laser detuning play remarkable roles. This resonance broadening by the *B*_1_ field is well known [16,33,46,47]. The weighting of the laser detuning is stronger for the medium-pressure cell, what once again reflects the more pronounced influence of the single absorption lines in this case.

The dependencies of the resonance depths are quite identical for both cells. For that reason, only the medium-pressure cell results are shown in Figure 14. All operational parameters play a role; however, the main contribution comes from the cell temperature. Its increase delivers more atoms to contribute to the signal, until the counteracting exponentially growing absorption in the cell impedes further signal improvement. 

The signal steepness of the difference signal is presented in Figure 15. Its performance relies on all other operational parameters. This inter-dependence is represented by the fact that all single CoP values add up to far more than 100%, indicating a lot of coupling terms between parameters in the approximation model. The stronger influence of the laser frequency detuning on many operational parameters in the medium-pressure cell, as seen before, is of course translated into the steepness dependence.

Figure 16 shows the CoP matrix of the shot-noise limited magnetic-field resolution *B*_sn_, where all other parameters act together. The total CoP is high, what means that we have reliable predictions, but all single CoPs are low and—moreover—partially remarkably different for the two cells. The reason can be seen in the curves within the colored squares: for every influence parameter there is a broad minimum of *B*_sn_. This means, that the values of all operational parameters are uncritical in a broad range. This is confirmed by the subsequent optimization of the operational parameters. The optimization results for the two cells are given in Table 1. 

Both optimization types deliver comparable results. Actually, for the medium-pressure cell they are identical. For the high-pressure cell the operational parameters are slightly different, what has only marginal influence on the value of the minimum noise, however. This once again underlines the insensitivity of *B*_sn_ to varying operational parameters. This conclusion can be drawn for the medium-pressure cell too. The measurements presented in Section 2.3. delivered a minimum of *B*_sn_ ≈ 9 fT/√Hz, what is analog to the optimized value of 8.5 fT/√Hz, even though the operational parameters were partially different (*T*_cell_ = 128 °C, *B*_1_ = 236 nT_rms_, *P*_L_ ≈ 1.6 mW). This even holds for the pump laser frequency. In the measurements of Section 2.3 we directly pumped the (buffer-gas shifted) F = 3 ground state. The optimizations show the best values to appear when the pumping laser frequency is slightly shifted towards the F = 4 ground state for both cells (cp. Figure 2). However, also this is not critical.

## 4. Conclusions and Outlook

An optically pumped magnetometer working in the new LSD-Mz mode was investigated and optimized by means of Latin Hypercube Sampling. The operation of the magnetometer was controlled and evaluated by the software. A complete, reliable, and physically plausible model of the magnetometer operation with the five operational parameters (pump laser power, pump laser frequency, cell temperature, *B*_1_-field amplitude, buffer gas pressure) could be obtained. In the result of a subsequent optimization, a shot-noise limited magnetic-field resolution *B*_sn_ below 10 fT/√Hz was achieved. This optimum is robust in regard to small input parameter variations. 

This shot-noise limited magnetic-field resolution is another step towards the ultimately obtainable intrinsic resolution *B*_in_ (given by Equation (1)) with such small vapor cell (*V* = 50 mm^3^) used in our integrated assemblies. At our working temperature of 128 °C, we recently determined the relaxation rates Γ/2π of the magnetometer cells to be about 1700 Hz and 50 Hz with and without spin-exchange relaxation, respectively [48]. With the cesium vapor density *n* = 7.5 × 10^13^ cm^−3^ resulting at this temperature, the intrinsic noise-limited magnetic-field resolutions *B*_in_ are 2.4 and 0.4 fT/√Hz, respectively. 

The obtainable resolution may be impaired by technical noise sources from the pump laser [49]. However, the LSD-Mz principle to subtract the signals of two identical vapor cells pumped with the same laser source inherently offers the possibility to eliminate technical laser noise from the signal [25,31]. For the light-shift noise such subtraction of two oppositely pumped cells is a common way to evade back-action in optical magnetometry [50]. Investigations on this topic will be a task for the near future.

Better shot-noise limited magnetic field resolutions (in the 1 fT/√Hz range for active cell volumes of about 100 mm^3^) are presently only achieved with magnetometers working in the SERF mode [28,51,52,53]. However, these magnetometers can only work in near-zero ambient magnetic fields, whereas the LSD-Mz magnetometer operates at Earth’s field strength (about *B*_0_ = 50 μT). So, the LSD-Mz-Magnetometer is a very promising candidate especially for geomagnetic prospection, but may also get interesting for biomagnetic measurements outside of magnetic shielding. 

## Figures and Tables

**Table 1:** Optimized shot-noise limited magnetic-field resolution *B*_sn_ with the parameters of the optimization procedure (upper part) and the corresponding operational parameters (lower part).

	High-Pressure Cell		Medium-Pressure Cell	
Optimization type	Global	Local	Global	Local
Algorithm	ARSM	simplex	ARSM	simplex
Measured designs	220	160	220	180
Measurement time [h]	7	4	7	4
**Minimum *B*_sn_ [fT/√Hz]**	**11**	**10**	**8.5**	**8.5**
*B*_1_-field amplitude [mV_rms_]	495	785	945	945
[nT_rms_]	172	272	328	328
Laser pump power [mW]	1.7	2.25	3.3	3.3
Cell temperature [°C]	130	128	130	130
Detuning voltage [mV]^1^	−55	−52	−90	−90
Detuned laser frequency [GHz]^2^	6.13	6.10	6.47	6.47

^1^ Detuning of the pump laser current driver. ^2^ Resulting detuning of the laser frequency from F = 4 → F’ = 3 vacuum transition.
